# Polyaniline-gold nanoparticle-based biosensor targeting miR-21-5p for early diagnosis of leptospirosis

**DOI:** 10.1007/s00216-026-06754-1

**Published:** 2026-08-25

**Authors:** Palanisamy Bothammal, Singarayan Michelraj, Ashutosh Verma, Ganesh Venkatachalam, Kalimuthusamy Natarajaseenivasan

**Affiliations:** 1 Medical Microbiology Laboratory, Department of Microbiology, Center for Excellence in Life Sciences, Bharathidasan University, Tiruchirappalli, 620 024 Tamil Nadu India; 2 Electrodics and Electrocatalysis (EEC) Division, CSIR – Central Electrochemical Research Institute (CSIR – CECRI), Karaikudi, 630003 Tamil Nadu India; 3 Lincoln Memorial University, College of Veterinary Medicine, Harrogate, TN 37752 USA; 4 Department of Neuroscience, Rowan-Virtua School of Osteopathic Medicine, Rowan University, Stratford, NJ 08084 USA

**Keywords:** Gold nanoparticle, MicroRNA, Electrochemical impedance spectroscopy, Leptospirosis, Early diagnosis

## Abstract

Blood-based disease diagnosis facilitates the early-stage detection of leptospirosis because leptospires were present in the blood in the first 5 to 10 days after exposure. We present a sensor employing polyaniline (PANI) and gold nanoparticles (AuNPs) for the specific detection of miR-21-5p in individuals with leptospirosis. The electropolymerization of PANI on glassy carbon electrodes (GCEs) creates a porous and durable coating that remains stable over extended periods without mechanical damage. To enhance the biosensor’s performance, we functionalized the sensor surface with gold nanoparticles through electrodeposition, which facilitated an electroactive surface and selective binding of the thiolated probe. Our data confirmed that the thiolated DNA probe (anti-miRNA-21) exhibits a higher affinity for the target miRNA-21 and serum samples from individuals with leptospirosis compared to non-complementary sequences and healthy serum samples. The biosensor demonstrated remarkable sensitivity with a detection range spanning from 1 aM to 50 nM, boasting a limit of detection (LOD) of 1 aM and a limit of quantification (LOQ) of 4.59 aM. This represents an unprecedented achievement in ultra-low-level miRNA-based biosensors for diagnosing leptospirosis. Early-stage disease detection is crucial for effective disease management and timely treatment. Such an approach holds promise for point-of-care testing in remote settings to facilitate the early diagnosis of leptospirosis.

**Graphical abstract:**

**Figure:**
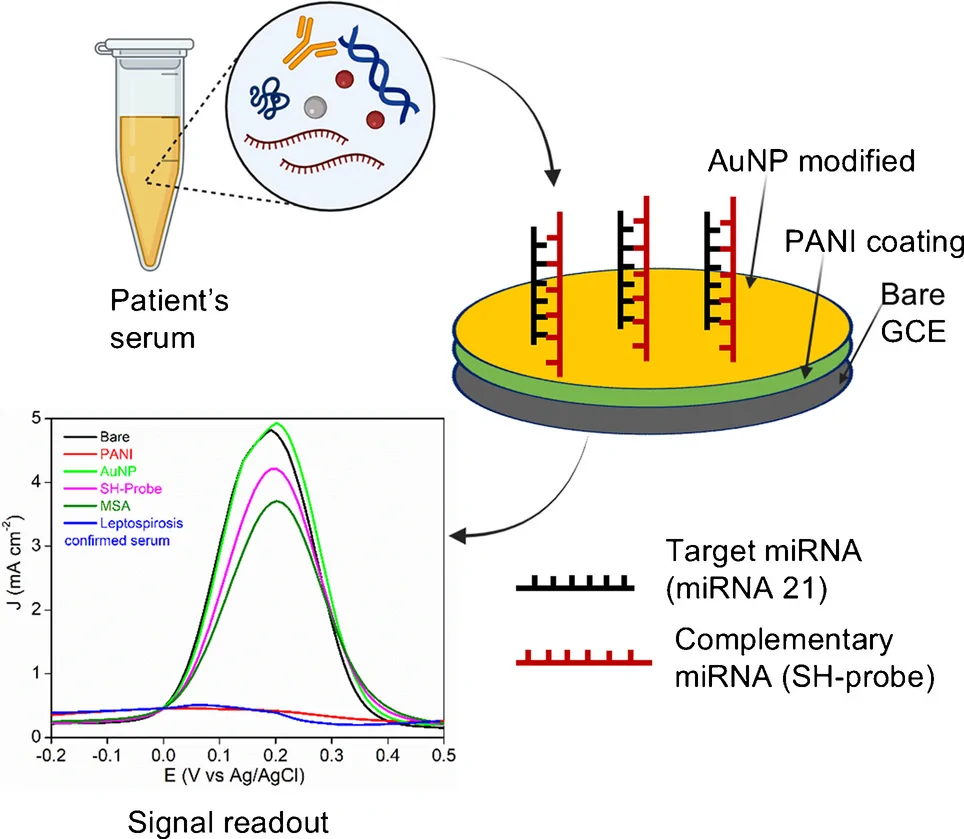

**Supplementary Information:**

The online version contains supplementary material available at https://doi.org/10.1007/s00216-026-06754-1.

## Introduction

Addressing the formidable challenges today is closely linked to the surging global population and escalating health concerns and ailments. Depending on the nature of the infection, the host organism may trigger an immune response, generating antibodies, hormones, or proteins to combat the invading pathogen. These vital biomolecules can be found in bodily fluids, such as blood, and have the potential to serve as crucial markers for disease detection, either as complete molecules or through their DNA/RNA signatures. Timely detection during the acute phase of an infection is imperative to curb its transmission, facilitate therapeutic interventions, and, in turn, reduce associated costs and manage the outbreak effectively. Despite the availability of various diagnostic tools for disease detection, many of them still grapple with certain limitations. As research delves into the emerging prognostic value of microRNAs, there is a growing focus on leveraging miRNAs as a diagnostic tool for point-of-care (POC) analysis.

MicroRNAs, commonly referred to as miRNAs, constitute a prevalent category of noncoding genes characterized by their small size, typically composed of approximately 22 nucleotides. They play a pivotal role in the regulation of gene expression. In various types of cancer, miRNAs exhibit both upregulation and downregulation [1–3]. In human cells, there are more than a thousand distinct microRNAs, with miR-21 emerging as one of the most consistently upregulated miRNAs across a spectrum of human cancer types, including breast, gastric, colon, lung, pancreatic, and ovarian cancers. miR-21’s influence extends to the promotion of disease progression through its ability to target tumor suppressor genes [4–8]. Notably, an abnormal expression of miR-21 has been identified in glioblastoma (GBM), an extremely aggressive form of brain tumor [9]. For instance, miR-155 and miR-21 exhibit upregulation in breast cancer [10, 11]. Beyond cancer, miR-21 also experiences upregulation in the context of various viral infections, which can lead to disruptions in the host’s immune system and promote viral replication. Conversely, miR-145 demonstrates downregulation in multiple cancer types, including bladder cancer, colon cancer, breast cancer, ovarian cancer, and prostate cancer [12, 13]. In breast cancer, miR-91 shows a downregulated pattern. It is noteworthy that miRNAs are present in bodily fluids such as blood serum, plasma, urine, and saliva [14, 15]. Circulating miRNAs maintain high stability in blood, often associated with the RNA-binding protein Nucleophosmin 1 or found within microparticles and exosomes [16, 17]. In recent years, several biosensors have been developed utilizing the major outer membrane protein LipL32 of *Leptospira* as a diagnostic target. While LipL32-based assays are specific and widely used, they primarily rely on host antibody production against the antigen. As a result, detectable antibody levels typically appear only after 5–7 days of infection, limiting their utility for early diagnosis [18–21]. In contrast, a microRNA (miRNA)-based diagnostic approach may offer significant advantages for early detection. miRNAs are released into circulation during the early stages of infection and can reflect host-pathogen interactions before a measurable humoral immune response develops. Because they are stable in body fluids and can be detected directly using molecular platforms, miRNA biomarkers have the potential to enable earlier and more sensitive diagnosis of leptospirosis.

In recent years, the occurrence of leptospirosis outbreaks has become an annual occurrence attributed to the impact of global climate change and increased flooding. Consequently, there is an urgent requirement to develop straightforward, cost-effective, non-invasive, and point-of-care testing methods for detecting leptospirosis in remote areas. Among the various biomarkers used for leptospirosis detection, serum biomarkers have demonstrated exceptional sensitivity. Previous research has identified three specific miRNAs (miR-let-7b-5p, miR-144-3p, and miR-21-5p) that exhibit elevated levels in response to exposure to leptospiral lipopolysaccharide [22].

In this study, we have engineered an exceptionally sensitive and selective electrochemical biosensor for the detection of miR-21-5p in individuals infected with leptospirosis. Circulating miR-21-5p has emerged as a promising biomarker owing to its stability in biological fluids and its dysregulated expression in a variety of pathological conditions [23, 24]. The abundance of miR-21-5p in serum and plasma can vary considerably depending on disease status, stage of progression, and sample processing methodology [25]. Consequently, sensitive and selective analytical platforms are required for its reliable quantification in clinical specimens. Although clinically relevant concentrations are generally higher than the attomolar range, the development of ultra-sensitive biosensors offers several advantages, including improved detection of low-abundance targets, enhanced analytical confidence following sample dilution, and the ability to monitor subtle changes in biomarker expression during the early stages of disease [26–28]. Therefore, achieving a very low limit of detection broadens the applicability of biosensing platforms and strengthens their potential utility in clinical diagnostics. To achieve this, we employed a PANI-AuNP-modified GCE for our sensing application. The utilization of conducting polymers has gained significant attention due to their superior electrical conductivity, strong adhesion properties, and excellent stability [29]. PANI was electrodeposited onto the GCE surface by cyclic voltammetry (CV), producing a porous and robust coating that facilitates efficient electron transfer [30, 31]. To further improve sensor performance, gold nanoparticles were subsequently electrodeposited onto the PANI-modified surface, providing a simple and cost-effective alternative to conventional chemical synthesis methods [32, 33]. In addition to enhancing the electroactive surface area, the AuNPs enable the stable immobilization of thiolated DNA probes through Au–S interactions. The synergistic combination of PANI and AuNPs contributes to the high analytical sensitivity of the developed biosensor for miR-21-5p detection.

## Materials and methods

### Apparatus and reagents

The electrochemical measurements were performed with a potentiostat/galvanostat from Metrohm Autolab, PGSTAT320N, controlled by NOVA 1.11 software. The three-electrode system consists of (i) a glassy carbon electrode as the working electrode (3 mm diameter), (ii) silver/silver chloride as the reference electrode, and (iii) platinum wire as the counter electrode. Ultrapure Milli-Q water (18.0 Ωm) or 0.1% diethylpyrocarbonate (DEPC)–treated autoclaved water was used for buffer preparation and in order to increase the stability of the miRNA by decreasing the RNase activity. All the reagents were used without further purification. The oligonucleotide probes were thiol-modified anti-miRNA-21 (complementary to target miRNA-21) and miRNA-21. Single mismatches and non-complementary sequences were obtained from Invitrogen. The sequences were as follows: (anti-miRNA-21) SH-TCA ACA TCA GTC TGA TAA GCT A, (miRNA-21) UAG CUU AUG AGA CUG AUG UUG A, (Single Mismatch) UAG CUU AUG AGA CUG AUG UUC A, and (non-complementary) UUC CUC GAC UGA CUG GGC UAG C. The stock solution of oligonucleotide (anti-miRNA) was prepared in SSPE buffer (0.02 M EDTA, 2.98 M NaCl, and 0.2 M phosphate buffer). The stock solution of miRNA-21 was prepared in Tris–Cl (0.02 M Tris, 0.14 M NaCl, 0.001 M MgCl_2_, 0.005 M KCl, and 0.001 M CaCl_2_ (pH 7.4)).

### Fabrication of electrochemical biosensor

#### Electropolymerization of aniline and electrodeposition of gold nanoparticles on the GCE surface

Prior formation of the monolayer or addition, the GCE surface was cleaned by washing with distilled water, followed by CV electrochemical treatment with 0.5 M H_2_SO_4_ for 15 cycles (50 mV/s; −0.1 to 1.5 V). Then, the GCE surfaces were thoroughly polished with 0.3 and 0.05 µm α-Al_2_O_3_ powders on a soft cloth and were sonicated with double-distilled water for 5 min to remove possible contaminants. Finally, the GCE surface was washed with ultrapure water. The PANI coating was formed on GCE surfaces by dipping the electrode in 0.5 M H_2_SO_4_ electrolyte containing 0.1 M aniline monomers and processing CV from −0.2 to 1.2 V at a scan rate of 50 mV/s for 10 cycles. After the polymerization of PANI, the fabricated PANI/GCEs were washed with double-distilled water to remove unpolymerized aniline monomers remaining in the PANI coatings, if any. The resulting GCE-PANI electrode was further modified with electrodeposition of gold nanoparticles. The GCE-PANI-AuNP electrode was obtained by CV scanning from 1.5 to −0.1 V in 0.5 M H_2_SO_4_ electrolyte containing 0.25 mM HAuCl_4_ at a scan rate of 50 mV/s. The modified electrode was washed with double-distilled water and dried under N_2_ atmosphere.

#### Immobilization SH-DNA probe (anti-miRNA-21) on the GCE-PANI-AuNP sensor

Further, the sensor area was modified with the thiolated DNA probe (anti-miRNA-21) complementary to the target miRNA-21. This was achieved by dipping the electroactive surface into the 1 nM solution for –SH DNA probe solution for 20 min at room temperature. After that, the surface was cleaned with double-distilled water to remove the unbinding probe from the surface. In order to achieve the selective binding, the surface area was blocked with mercaptosuccinic acid (MSA) (0.01%) for 20 min, which increased the selective binding of the further target analyte to the probe, not with the surface. Blocking AuNP-based biosensors with MSA is an effective strategy to enhance selectivity by creating a stable, negatively charged carboxylic acid monolayer that prevents non-specific adsorption. MSA acts as a strong, thiol-based capping agent that forms a self-assembled monolayer (SAM) on the gold surface [34].

#### Hybridization of target analyte (miRNA-21) to the GCE-PANI-AuNP sensor

To achieve the goal of our work, the sensor area was subjected to hybridization with our target miRNA-21. The electrode was dipped into different concentrations of miRNA solution for about 20 min. For real sample analysis, the sensor area was dipped into leptospirosis-confirmed serum samples. Serum samples (Infected/healthy) were diluted in PBST (1:50 dilution) and incubated for about 20 min at room temperature. Similarly, the sensor selectivity was achieved using single-mismatch RNA, non-complementary RNA, and healthy serum samples.

### Electrochemical analysis

All the electrochemical experiments were recorded using the AUTOLAB PG STAT instrument. The electrical properties before and after modification of GCE surfaces were determined by three electrochemical analyses, namely CV, electrochemical impedance spectroscopy (EIS), and square wave voltammetry (SWV). All the electrochemical characterization was performed using the redox probe solution 5.0 mM of K_3_ Fe(CN)_6_ and K_4_Fe(CN)_6_ prepared in PBS buffer (pH 7.4)_._ CV analysis was recorded with the potential between −0.2 and 0.6 V at a scan rate of 30 mV/s. EIS assays were performed between the frequencies of 0.1 and 100,000 Hz with the reference potential of 0.2 V, using a sinusoidal potential perturbation with an amplitude of 0.05 V and 60 data points. Then, the EIS data were fitted into Randle’s equivalent circuit, using Zsimpwin software. SWV experiments were conducted between the potential range of −0.2 and 0.8 V, with a frequency of 20 Hz and step height of 0.05 V.

## Results

### Biosensor fabrication process

**Scheme 1:**
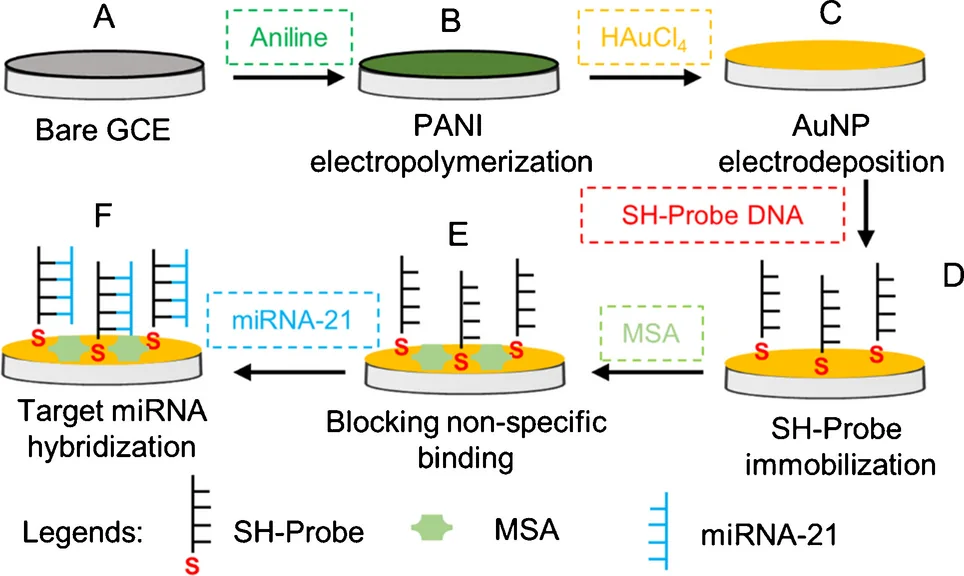
Schematic illustration of biosensor fabrication process: **A** GCE electrode pretreatment; **B** Electro polymerization of PANI; **C** In situ gold nanoparticle electro polymerization; **D** Immobilization of probe DNA (antimiRNA 21); E Non-specific blocking with Mercaptosuccinic acid and **F** Target (miRNA 21) RNA binding

The schematic illustration of biosensor assembly is shown in Scheme 1. The steps involved in biosensor construction were (1) GCE electrode pretreatment, (2) electropolymerization of PANI, (3) in situ gold nanoparticle electropolymerization, (4) immobilization of probe DNA (anti-miRNA-21), (5) non-specific blocking, and (6) target (miRNA-21) RNA binding.

Initially, the GCE electrode was cleaned by polishing with alumina slurry to remove any contaminants on the electrode surface and to achieve a clean, smooth surface. To ensure a clean electrode, the surface was washed thoroughly with distilled water. Further, to remove any organic or inorganic contaminants on the electrode surface, electrochemical cleaning was performed by CV analysis using an H_2_SO_4_ electrolyte. Secondly, the PANI films were electropolymerized in the presence of 0.1 M aniline in 0.5 M H_2_SO_4_. The voltammograms measured during the synthesis of the PANI coatings on the surface of GCE are shown in Fig. S1A. In the CV, the oxidation peaks at 0.235 V indicate the transformation of the leucoemeraldine form (fully reduced state) to the emeraldine salt form (neutral salt). Then, the oxidation peaks at about 0.513 V refer to the state transformation from emeraldine form to pernigraniline form (fully oxidized state). The oxidation peaks at about 0.706 V are related to the polymerization of aniline monomers, which confirms PANI polymerization.

In the third step, to increase the sensitivity of the electrode surface by in situ electrodeposition on the PANI-modified GCE using 0.1 M H_2_SO_4_ electrolyte containing 0.25 mM of HAuCl_4_ through 20 cycles of CV analysis of a potential range of 1.5 to 0.1 V at a scan rate of 50 mV/s (Fig. S1B). Anodic peak current at 1.3 V is associated with the Au oxidation, and in the backward scan, the peak at 0.89 V is the subsequent reduction of oxides previously formed. Then, in the fourth step, to functionalize the GCE-PANI-AuNP surface, the electrode was subjected to immobilization of SH-probe DNA (anti-miRNA-21), by dipping the modified electrode into 10 nM of probe DNA solution for about 20 min. The probe DNA was covalently bonded with AuNP on the GCE-PANI electrode with its thiol group (–SH) in the 5′ end. It had the complementary sequence to the target analyte miRNA-21. Next, in the fifth step, to enhance the specificity, the electrode surface was blocked with MSA because its –SH bond allows it to covalently bind with free AuNP areas on the modified electrode. This step was performed in order to avoid direct interaction or binding of the target analyte or any other biomolecule to the GCE-PANI-AuNP electrode [11]. Finally, the hybridization of target miRNA-21 to the probe DNA was performed by incubating the modified working electrode into the solution of different concentrations of target miRNA for about 20 min at room temperature.

### Electrochemical characterization of a modified sensor

**Fig. 1:**
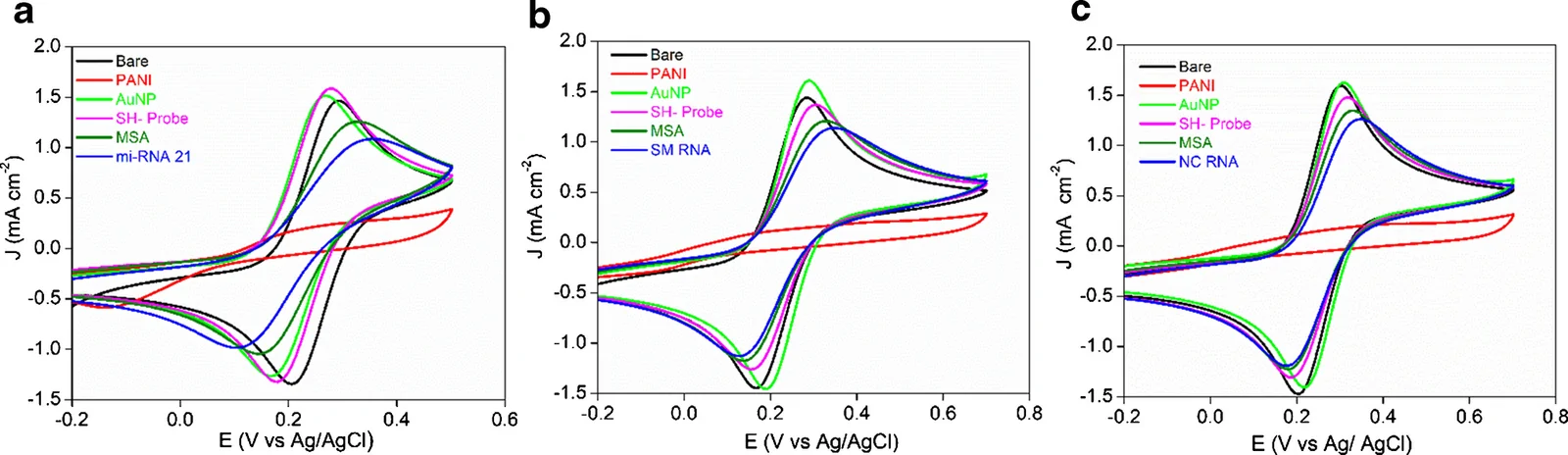
Cyclic voltammogram of different modifications of the sensor. **a** CV analysis of the sensor for target miRNA-21. **b** Single mismatched RNA. **c** Non-complementary RNA

Chemical modification of the biosensor surface was electrochemically evaluated using different parameters like CV, EIS, and SWV. The chemical species in each modification produced alterations in its electrical features, and these electrical properties were identified by using the standard redox probe Fe [(CN)_6_]^3−/4−^ in PBS (pH 7.4). As shown in the CV analysis in Fig. 1, when compared with bare GCE electrode, the GCE-PANI-AuNP modified with probe, MSA, and miRNA-21 showed decreased cathodic/anodic peak currents because the surface becomes more engaged after each modification that hardens the redox probe to access the electrode surface for the electron transfer process. Further, the increased potential separation was noticed due to the introduction of insulating layers on the surface which hinders the efficient movement of charge carriers. This confirms the increased charge transfer resistance after modification on the working electrode. EIS measurements provide more information on charge transfer resistance and the double-layer capacitance. After modification, increase in the charge transfer resistance was observed, consistent with the CV data. In addition, the SWV data also showed a decreased peak current after surface modification which further confirms the successful immobilization of the probe DNA, MSA, and miRNA-21 on the electrode surface. Similarly, the corresponding EIS measurements and SWV data were consistent with CV data.

**Fig. 2:**
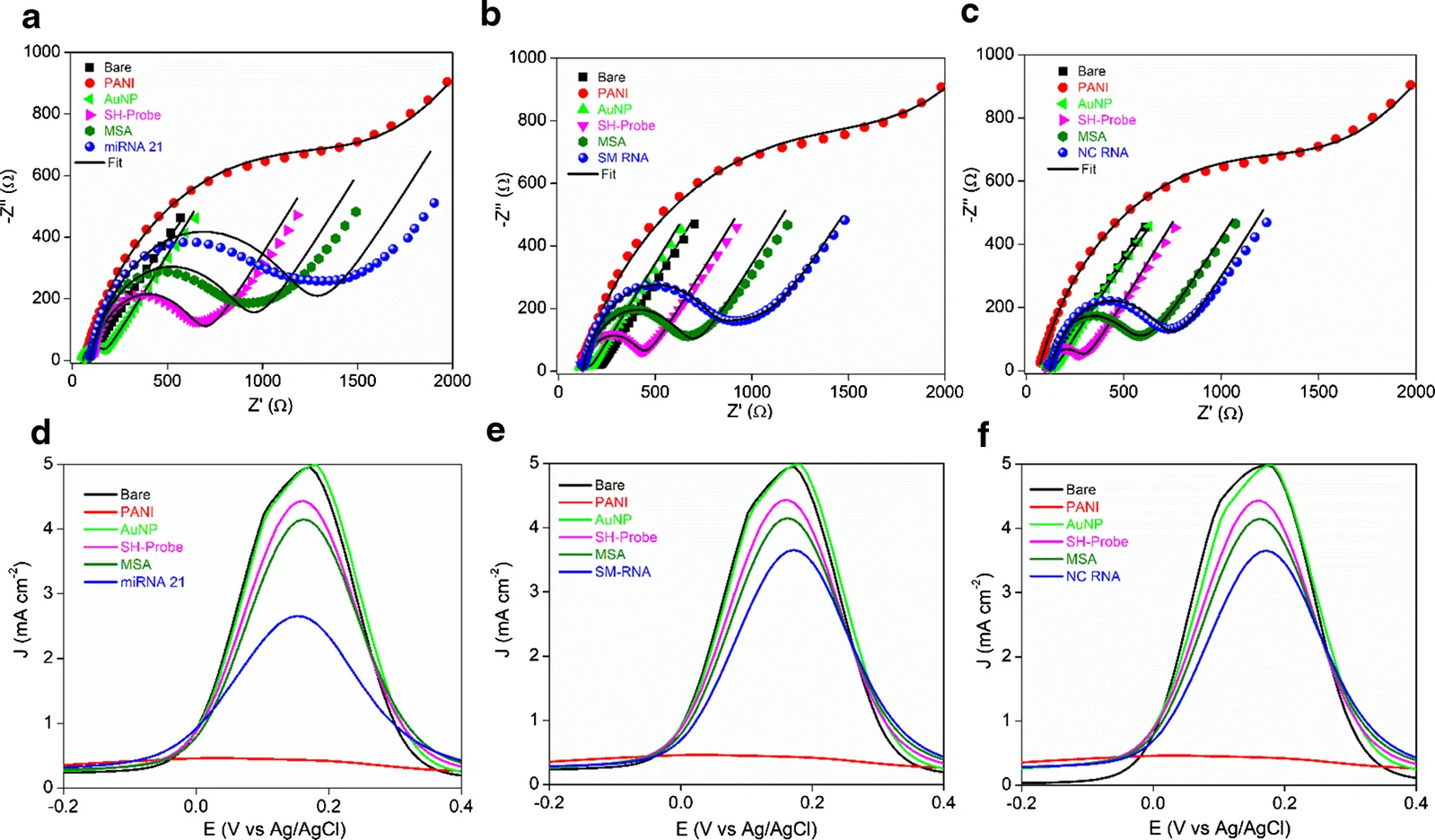
EIS and SWV analysis of modified sensor. **a**–**c** Nyquist plot and (**d**–**f**) SWV of modified sensor for target miRNA-21, single mismatched RNA, and non-complementary RNA

A Nyquist plot is an effective way to measure the electron-transfer resistance on the electrode surface. Randle’s equivalent circuit was used to fit the physicochemical process occurring at the working electrode surface. In the Nyquist plot, the semicircle indicates the electron transfer resistance (*R*_CT_), and the linear part represents the diffusion-limited process. We used Randle’s equivalent circuit R(Q(RW)) and modified one in some cases (Fig. S2). It provides the magnitude of the electrolyte solution resistance (Rs), the charge transfer resistance (*R*_CT_) is inversely proportional to the rate of electron transfer, and the Warburg diffusion element (*W*) at higher frequencies relates to the diffusion of ions. The Nyquist plot of each modification is shown in Fig. 2a–c. The electron transfer resistance (*R*_CT_) of the bare electrode, PANI-coated, AuNP-modified, probe DNA immobilization, and MSA blocking were 85, 1723, 104, 569, and 819 Ω, respectively. After hybridization with target miRNA-21, the electron transfer resistance was drastically increased to 3085 Ω. Furthermore, the probe DNA had less *R*_CT_ when incubated with single-mismatch and non-complementary RNA (1137 and 778 Ω) compared to the target miRNA-21. After modification on the working electrode, the diameter of the semicircle increased, which indicates the increased electron transfer resistance at the surface, and the results were consistent with the CV data. The different elements present in the Nyquist plot at each modification are presented in Table 1.

**Table 1:** The different elements present in the Nyquist plot in the different modification of the sensor

	***R***_S_ (Ω cm^2^)	CPE (10^–6^)	Freq (Hz)	***R***_CT_ (Ω cm^2^)	Warburg	Ch.Sq (10^–4^)
Bare	29.69	6.12	0.820	85	0.0016	4.09
PANI	31.79	4.31	0.694	1723	5.9 × 10^–5^	2.92
AuNP	36.36	6.5	0.776	104	0.0018	4.34
Probe DNA	97.42	1.52	0.893	569	0.0017	1.56
MSA	89.7	7.2	0.863	819	0.0015	1.92
miRNA-21	91.02	4.05	0.865	3085	0.0012	1.36
SM RNA	93.57	5.03	0.798	1137	0.0018	1.73
NC RNA	112	4.03	0.847	778	0.0013	2.6
Lepto serum	109	3.2	0.121	8.9(× 10^27^)	9.306	4.3
Healthy serum	106	3.2	0.815	529	0.0017	1.45

SWV is considered more sensitive than CV; the changes in the electrical properties after each addition and the decrease in the peak current were observed in the voltammograms. Figure 2d–f show the SWV of bare electrode and sub sequential modification. Initially, the bare electrode had the peak current density of 4.965 mA cm^−2^. There was a significant reduction in the peak current density observed after polymerization with PANI (0.439 mA cm^−2^). It was noted that the in situ polymerization of gold nanoparticles facilitated the electron transfer on the surface with the current density of 4.980 mA cm^−2^. However, the sequential immobilization of the electrode with probe DNA results in the reduction of peak current to 4.435 mA cm^−2^, confirms the AuNP, and anchors the probe DNA by creating the covalent bond interaction. By blocking MSA, the surface had a reduced peak current of 4.148 mA cm^−2^. After hybridization with target miRNA-21, a significant reduction in the peak current to 2.656 mA cm^−2^ confirms the effectiveness of the sensor. Moreover, incubation with non-specific (3.653 mA cm^−2^) or non-complementary RNA (3.651 mA cm^−2^) also has the reduction in the peak current, however showing non-significant binding in comparison to target miRNA-21. Table 2 represents the potential and current density results of the different modifications on the sensor electrode. Together, these assays were consistent and confirmed the changes in the electron transfer resistance on the electrode surface.

**Table 2:** Potential and current density results of the different modifications on the sensor electrode

	Potential (V)	Current density (mA cm^−2^)
Bare	0.161	4.965
PANI	0.142	0.439
AuNP	0.172	4.980
Probe DNA	0.161	4.435
MSA	0.162	4.148
miRNA-21	0.151	2.6560
SM-RNA	0.172	3.653
NC-RNA	0.172	3.651
Lepto serum	0.1919	0.3998
Healthy serum	0.202	3.649

### Analytical performance of the biosensor

**Fig. 3:**
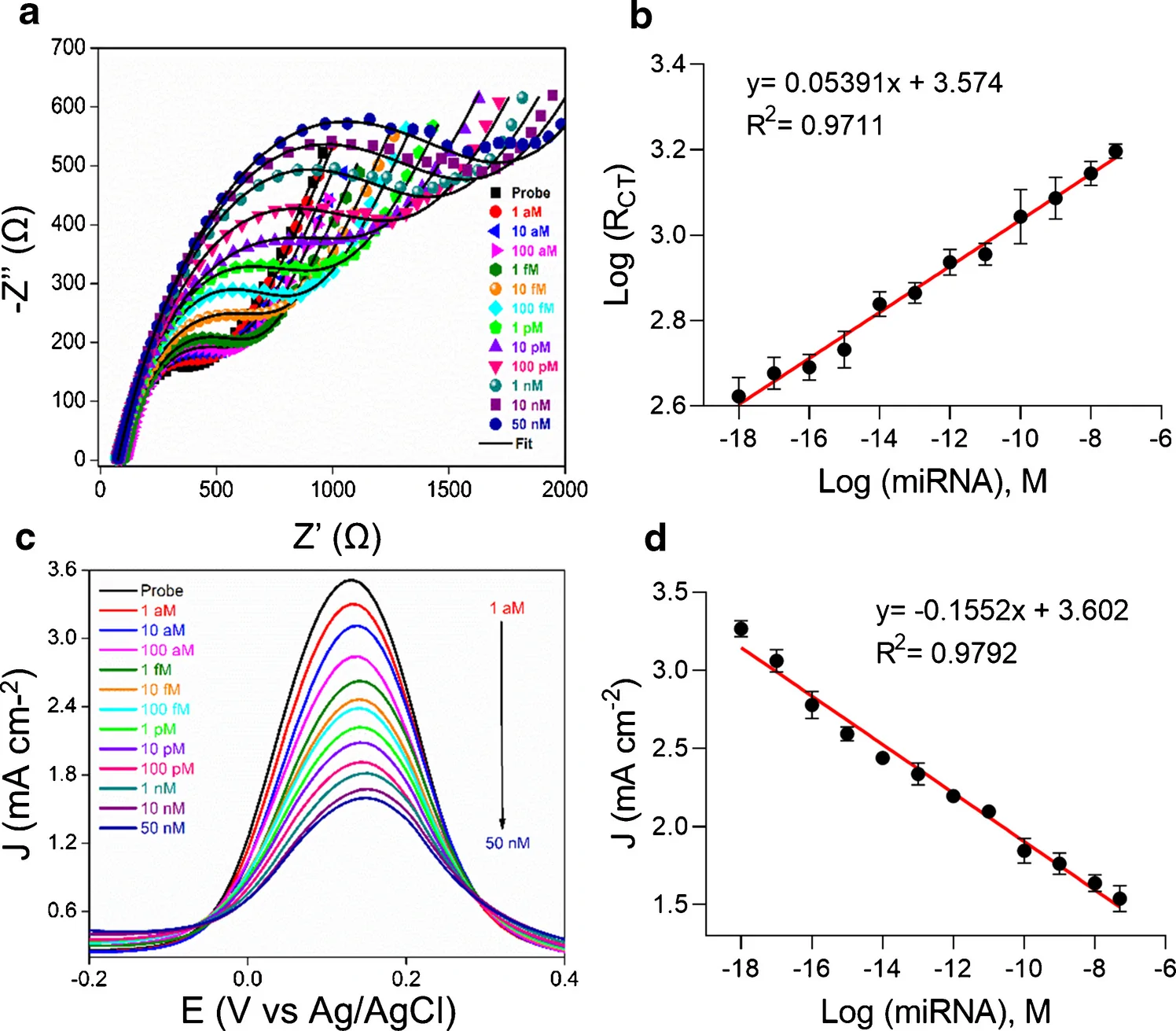
miRNA concentration optimization. **a** Nyquist plot of various concentrations of miRNA (1 aM to 50 nM). **b** Linear plot against the miRNA concentration vs log (RCT). **c** SWV voltammogram. **d** Their linear plot against current density vs miRNA concentration

The concentration of miRNA was determined by different electrochemical experiments, such as EIS and SWV. For this purpose, we selected a concentration range between 1 aM and 50 nM of miRNA, and during each interval, the sensor was incubated for 20 min. Figure 3a represents the EIS responses of different concentrations of miRNA. The electron transfer resistance (*R*_CT_) value for 1 aM, 10 aM, 100 aM, 1fM, 10 fM, 100 fM, 1 pM, 10 pM, 100 pM, 1 nM, 10 nM, and 50 nM were 451, 505, 516, 579, 723, 762, 908, 941, 1226, 1323, 1463, and 1617 Ω, respectively. It can be seen that an increase in the *R*_CT_ upon increasing the concentration confirms that more charge transfer reactions occur at the surface-active area. The linear relationship between the electron transfer resistance and the logarithmic value of miRNA concentrations from 1 aM to 50 nM, with the slope of EIS, was found to be 0.0539, the *R*^2^ was determined as 0.9711 (Fig. 3b), and the elements present in this Nyquist plot and their values are indicated in Table S1. The limit of detection (LOD) was determined using the formula 3(*σ*/*S*), and the limit of quantification (LOQ) was calculated as 10(*σ*/*S*), where *σ* represents the standard deviation of the blank (or the lowest concentration) and *S* is the slope of the calibration curve. Based on this, the LOD and LOQ were approximately 1 aM and 4.5 aM, respectively. Similarly, the square wave voltammogram is consistent with the Nyquist plot, whereas, in SWV, the current density was decreased accordingly as the concentration dose (Fig. 3c). The observed peak currents were 3.2302, 3.0114, 2.7184, 2.5626, 2.4150, 2.2870, 2.1719, 2.1085, 1.7892, 1.7156, 1.6003, and 1.4814 mA cm^−2^ for 1 aM, 10 aM, 100 aM, 1fM, 10 fM, 100 fM, 1 pM, 10 pM, 100 pM, 1 nM, 10 nM, and 50 nM level of miRNA and having the slope and *R*^2^ values were −0.1552 and 0.9792, respectively (Fig. 3d). Overall, the biosensor exhibited a wide linear detection range (1 aM–50 nM) with strong correlation coefficients in both EIS and SWV analyses, confirming reliable and concentration-dependent responses. Table 3 summarizes and compares the sensor platforms, detection methods, linear detection ranges, and limits of detection (LODs) of previously reported biosensing strategies with the present biosensor. The proposed biosensor demonstrates high sensitivity and a broad linear detection range comparable to those of previously reported platforms.

**Table 3:** Comparison of selected methods for detection of miRNA-21

Sensor platform	Analytical detection strategy	Linear range	LOD	Reference
Chemiluminescence (CL) biosensor based on CRISPR-Cas12a and cation exchange reaction	ECL	Not mentioned	16 M	[59]
Gold platinum bimetallic nanoparticles (AuPtBNPs)/3-aminopropyltriethoxy silane (APTS) nanocomposite coated fluorine-doped tin oxide (FTO)	DPV	1 fM–100 nM	0.63 fM	[60]
Electrochemical biosensor using catalytic hairpin assembly (CHA) and rolling circle amplification (RCA) as a dual signal amplification strategy	DPV	0.5–12,500 pmol	290 fmol	[61]
Sensor type: electrochemical biosensor - Components: graphene (GP), polypyrrole (PPY), gold nanoparticles (AuNPs), screen-printed carbon electrode (SPCE)	DPV	1.0 fM to 1.0 nM	0.020 fM	[62]
Gold nanoparticles modified pencil graphite electrode (PGE)	DPV	200 pM to 388 nM	100 pM	[63]
Electrogenerated chemiluminescence (ECL) biosensor with dual signal amplification based on isothermal strand-displacement polymerase reaction (ISDPR) and bridge DNA-gold nanoparticles (AuNPs) nanocomposites, fabricated by self-assembling a thiolated capture probe on a gold electrode	ECL	0.01 fM to 10,000 fM	3.2 aM	[64]
Electrochemical biosensor using carboxylate-reduced graphene oxide (COOH-rGO) and duplex-specific nuclease (DSN) on a gold electrode (GE) with capture DNA (cDNA) and electroactive methylene blue (MB) molecules	DPV, EIS	Not mentioned	0.01 fM	[65]
The sensor platform is based on multiwall carbon nanotubes/graphene oxide nanoribbons (MWCNTs/GONRs) with ssDNA capture probes immobilized on the surface of the MWCNTs@GONRs/AuNPs modified electrode through the Au–S bond, and includes streptavidin-conjugated alkaline phosphatase (SA-ALP) attached to the end of the probe	DPV, EIS	Not mentioned	0.034 fM	[66]
The sensor platform consists of a neutravidin-modified glassy carbon electrode and a dual-function bio-label made of biotinylated DNA/LNA molecular beacons conjugated with gold nanoparticles (AuNPs)	EIS	1–1000 pM	0.3 pM	[67]
Dendritic gold nanostructure (DenAu) and graphene nanosheets modified glassy carbon electrode	EIS, DPV	Not mentioned	0.06 pM	[68]
Polyaniline-gold nanoparticle-based biosensor	EIS, SWV	1 aM to 50 nM	1 aM	This work

### Clinical sample analysis

**Fig. 4:**
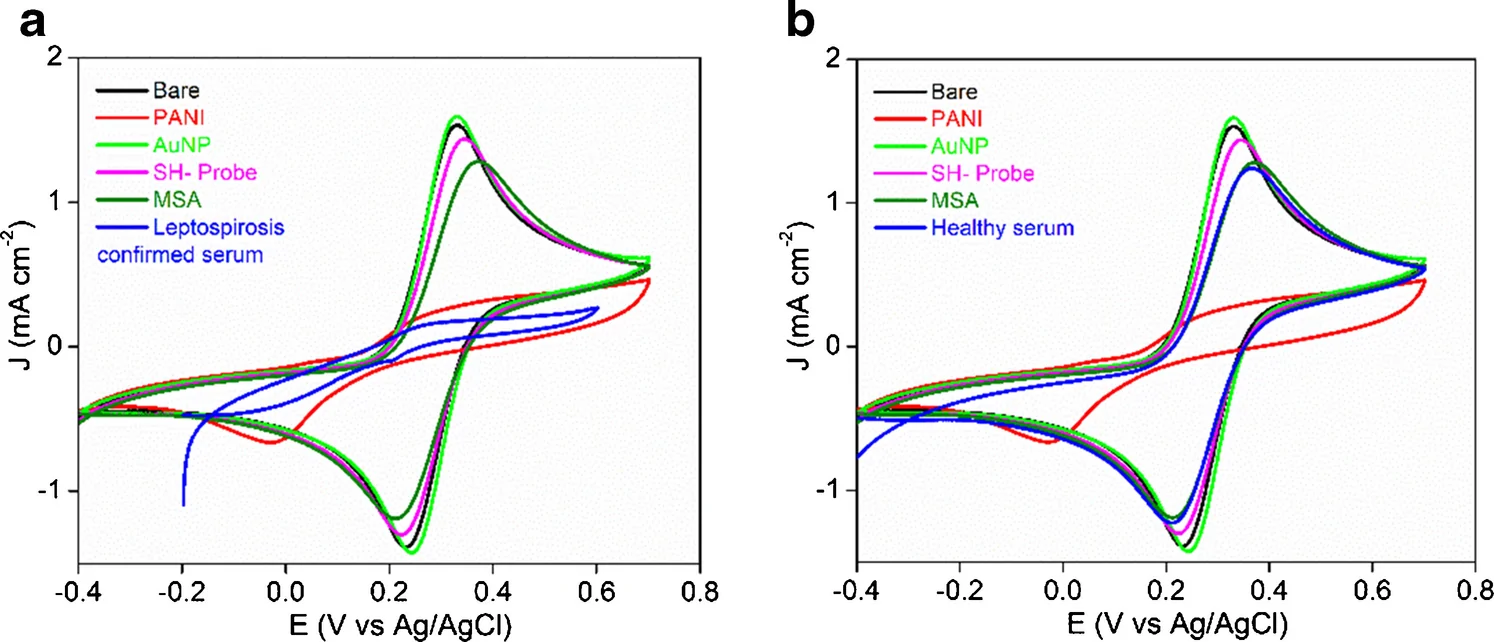
Cyclic voltammogram of the sensor selectivity. CV of (**a**) leptospirosis confirmed sera and (**b**) healthy serum sample analysis of the sensor electrode

The developed sensor was used for leptospirosis diagnosis using clinical samples. We assessed 5 clinical samples, where 3 leptospirosis-confirmed patient’s serum samples (MAT) and 2 healthy serum samples to demonstrate this sensing platform. The samples were obtained from KRS Multispecialty Hospital, Edappadi, Tamil Nadu, India. The studies involving human participants (for blood sample collection) were reviewed and approved by the Institutional Ethical Committee (No: DM/2014/101/51) of Bharathidasan University. After modification with the thiolated probe to the electroactive surface of the working electrode, the sensor electrode was dipped into the leptospirosis-confirmed patient’s serum or healthy serum and incubated for about 20 min. As shown in Fig. 4, a drastic decrease in the peak current to 0.3998 mA cm^−2^ as well as a shift in the peak potential to 0.1919 V was observed when compared to healthy serum having the peak current of 3.649 mA cm^−2^ and the probe DNA having a peak current of 4.435 mA cm^−2^.

**Fig. 5:**
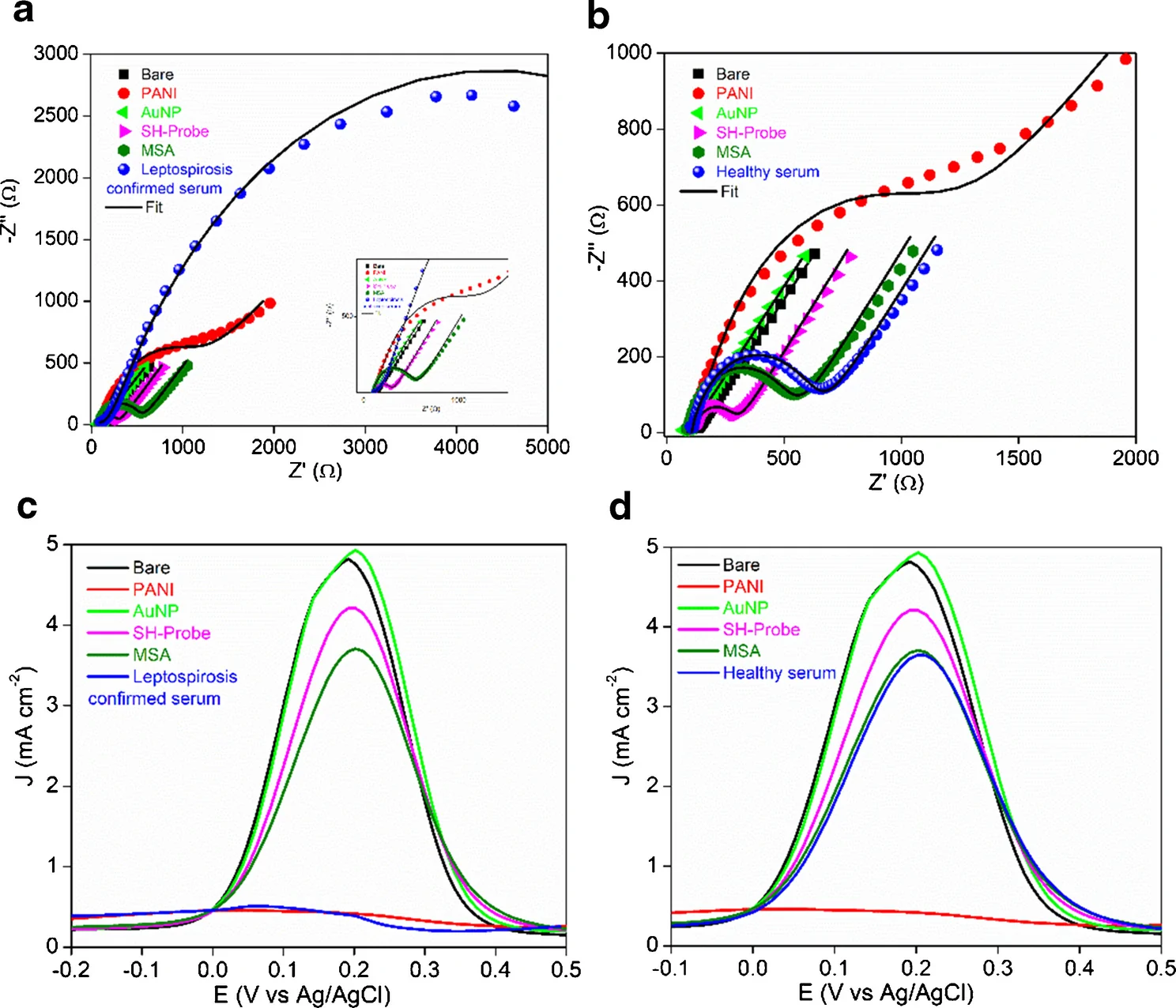
Nyquist plot and SWV spectrum of the sensor selectivity. **a** and **b** EIS spectrum and (**c** and **d**) SWV spectrum of leptospirosis confirmed sera and healthy serum sample analysis of the sensor electrode

In the EIS spectrum of the sensor against clinical samples, it indicates a drastic increase in the *R*_CT_ value of 8.9 × 10 ^27^ Ω in the leptospirosis serum-modified sensor and 529 Ω in the healthy serum (Fig. 5a and b). These results confirm that mass charge transfer occurs in the sensor surface area after incubation with leptospirosis-confirmed samples. The SWV results are also consistent with EIS and CV data, as shown in Fig. 5c and d, showing a drastic decrease in the current density when compared with healthy serum samples. The developed sensor can identify the leptospirosis-confirmed sera by a decrease in the peak current compared to the healthy control sera (Fig. S3). Overall, the developed sensor has been able to discriminate the leptospirosis cases from the healthy individual sera and confirms the specificity of the sensor.

## Discussion

MicroRNAs (miRNAs) stand out as highly dependable biomarkers extensively employed in biomedical diagnostics across a spectrum of ailments, encompassing various cancer types and infectious diseases. These miRNA-based biomarkers have been instrumental in early detection, diagnosis, metastasis assessment, prognosis determination, and evaluation of treatment effectiveness [35, 36]. miRNAs, constituting a class of small, non-coding endogenous RNAs approximately 22 nucleotides in length, play a pivotal role by exerting negative control over the expression of their target genes at the post-transcriptional level [37, 38]. Notably, a substantial exhibit of miRNAs, referred to as “circulating miRNAs,” exhibits remarkable stability when released into the bloodstream, with their expression levels exhibiting a specific correlation with disease stage, initiation of tumorigenesis, cancer progression, or metastasis [39, 40]. Given these advantageous features, we harnessed miRNA-based biomarkers for the detection of leptospirosis.

Various methods are currently employed for the quantification of miRNA due to their distinct advantages. These methods include real-time quantitative polymerase chain reaction (qPCR), Northern blotting, microarray analysis, and deep sequencing of the transcriptome (RNAseq) [39, 41]. Real-time qPCR is the most sensitive and commonly used technique for miRNA assessment, even in smaller laboratories. However, it is considered an advanced method that relies on expert knowledge, demanding expensive reagents and sophisticated equipment [42]. The traditional method of Northern blotting involves size-based separation of RNA samples and their subsequent hybridization with a complementary probe. This technique is limited by its low sensitivity, time-consuming process, and the use of hazardous radioactive labels [43, 44]. Microarray-based methods have substantial applications, allowing for the examination of a large number of samples simultaneously and generating extensive results. Despite its ease of use, microarray-based analysis is primarily a qualitative technique and lacks the essential sensitivity required for miRNA quantification [45, 46]. In contemporary research, deep sequencing of the transcriptome is widely employed to investigate miRNA in biological samples. This high-throughput technology enables parallel analyses and provides the capability to explore the expression of all annotated miRNAs [47, 48]. However, its primary application lies in the discovery of novel miRNAs rather than serving as a practical tool for miRNA quantification in diagnosis or screening purposes.

In the past decade, miRNA-based biosensors have emerged as a highly promising and captivating field of research. Recent advancements in nanoscale science and technology have made a significant impact on the biosensor landscape [49]. The integration of nanomaterials into the design and fabrication of biosensing systems not only reduces the overall device size but also imparts valuable enhancements, resulting in improved accuracy and reproducibility in bioanalysis results [50, 51]. The utilization of nanomaterials on electrode surfaces and electrochemical platforms in biosensor development represents an appealing strategy, primarily enhancing surface area, chemical stability, and the number of binding events [49, 52]. Leveraging the distinct advantages of nanomaterial-based electrochemical biosensors, we have created a miRNA biosensor for the detection of leptospirosis, employing gold nanoparticles as modifiers. PANI coatings have a porous and branched structure that can significantly increase the specific surface area, which enhances the electrochemical properties such as conductivity and charge storage. In situ electrodeposition of AuNPs over the PANI-modified GCE surface enhances the electrocatalytic behavior of the surface [53]. AuNP-modified electrode greatly facilitated the electron transfer kinetics compared to bare electrode [54]. The AuNPs offer excellent conductivity and biocompatibility, making the sensor surface ideal for biomolecule immobilization like RNA or DNA. CV, a popular electrochemical technique to investigate the oxidation and reduction processes of molecular species, also provides direct insights into the kinetics of electrode reactions including both heterogeneous and homogeneous electron transfer reactions and coupled chemical reactions [55, 56]. In our results, we found that after modification of the surface, more chemical species were occupied on the surface which corresponds to the decrease in the peak current and shift in the potential. EIS, a powerful technique, is employed to sense the chemical or biochemical reactions that occur on the electrode surface such as antigen-antibody interaction, biotin-avidin complexes, and oligonucleotide-DNA interaction [57]. It is important to note that EIS itself is not a selective detection technique; instead, it acts as a highly sensitive method for monitoring interfacial changes at the electrode surface. The analytical selectivity of the present biosensor is derived from the sequence-specific hybridization between the immobilized thiolated DNA probe and the target miR-21-5p. Hybridization leads to the formation of a negatively charged nucleic acid duplex on the electrode surface, which hinders the electron transfer of the [Fe(CN)₆]^3^⁻/^4^⁻ redox couple and increases the charge-transfer resistance. The significantly different responses obtained for complementary, single-base mismatch, and non-complementary sequences confirm that the observed impedance changes are predominantly associated with specific probe–target interactions rather than non-specific surface adsorption. As seen from the result, increases in the diameter after modification except AuNP modification in compared to bare electrode, indicating the electron transfer resistances increased with the increasing the miRNA concentration or modification of the surface. SWV is another electrochemical technique, where a staircase potential is modulated by a series of potential pulses. During the cathodic pulse, the analyte is reduced at the electrode surface. In the anodic pulse, the analyte that was just reduced undergoes oxidation. This cycling of reduction and oxidation allows for the assessment of both the reduction and oxidation characteristics of the analyte. The current is sampled before the pulse and at the end of the pulse. The current difference between these two time points is recorded and plotted against the staircase potential, resulting in a square wave voltammogram. SWV is faster and more efficient than EIS. While EIS may require a longer time to gather enough data (due to the need for low-frequency sweeps), SWV can complete a linear potential scan in minutes. Additionally, SWV generates a peak-shaped signal, which is easier to interpret for analytical purposes [58]. The SWV results obtained were in agreement with the EIS and CV data. Moreover, we evaluated the developed sensor against leptospirosis cases by laboratory-confirmed MAT and healthy individual sera. Our findings unequivocally demonstrate that this developed sensor exhibits a high degree of specificity and sensitivity in diagnosing leptospirosis. Despite the promising analytical performance of the developed biosensor, several limitations should be acknowledged. First, the clinical sample size used for evaluation was relatively limited, and future studies involving larger and more diverse patient cohorts are necessary to further validate the diagnostic performance and clinical utility of the platform. Although the developed biosensor demonstrated promising performance in differentiating leptospirosis-positive and healthy serum samples, the clinical validation performed in this study was limited to a small number of samples. Therefore, the findings should be considered preliminary and primarily serve as a proof-of-concept demonstration of the platform’s feasibility in a complex biological matrix. The limited cohort size restricts the statistical power of the analysis and does not permit a comprehensive evaluation of diagnostic sensitivity, specificity, or inter-individual variability. Furthermore, the current study did not include samples from patients with other infectious or inflammatory diseases that may exhibit altered miRNA expression profiles. Consequently, larger validation studies involving diverse patient populations and clinically relevant control groups will be required to establish the clinical robustness, diagnostic accuracy, and translational potential of the proposed biosensing platform. Second, although the selectivity of the biosensor was successfully demonstrated using a single-base mismatch and a non-complementary sequence, additional investigations employing double- and triple-base mismatch targets would provide a more comprehensive assessment of sequence discrimination and specificity. Finally, while the sensor showed satisfactory performance in diluted human serum samples, a systematic quantitative interference study involving individual serum constituents, such as proteins, metabolites, and non-target nucleic acids, was beyond the scope of the present work. Such studies would offer deeper insight into the robustness of the biosensor in complex biological matrices and will be an important focus of future investigations aimed at clinical translation.

## Conclusion

Our findings indicate that the miRNA-based biosensor we have developed exhibits a greater degree of selectivity for the target miRNA-21, which is significantly upregulated in individuals afflicted with leptospirosis. The data also confirms that the thiolated probe DNA (anti-miRNA-21) displays a higher affinity for both the target miRNA-21 and serum samples from leptospirosis-positive cases compared to non-complementary sequences and healthy serum samples. In summary, our developed sensor boasts a LOD of 1 attomole (aM) and a LOQ of 4.59 aM, making it an ultralow-level miRNA-based biosensor for the diagnosis of leptospirosis. However, this study is limited by the relatively small number of clinical samples, underscoring the need for validation with larger cohorts and evaluation against samples from patients with other infectious diseases and cancers.

## Supplementary Information

Below is the link to the electronic supplementary material.

ESM 1 Supplementary Material 1 (DOCX 351 KB)

## Data Availability

All relevant data supporting the findings of this study are available in the paper and its Supplementary Information files. Any additional data supporting the conclusions of this study are available from the corresponding author upon reasonable request.

## Abbreviations

PANI: Polyaniline
GCE: Glassy carbon electrode
DNA: Deoxyribonucleic acid
RNA: Ribonucleic acid
LOD: Limit of detection
miRNA: MicroRNA
CV: Cyclic voltammetry
EIS: Electrochemical impedance spectroscopy
SWV: Square wave voltammetry
POC: Point-of-care
MSA: Mercapto succinic acid
LOQ: Limit of quantification
DEPC: Diethyl pyrocarbon
AuNP: Gold nanoparticle
