# Supplementary material for: Polyaniline-gold nanoparticle-based biosensor targeting miR-21-5p for early diagnosis of leptospirosis
Source: Anal Bioanal Chem. 2026 Aug 25;418(22):7541–53. doi: 10.1007/s00216-026-06754-1 (PMC13736323; doi:10.1007/s00216-026-06754-1)
Supplement: Supplementary file 1 [file 216_2026_6754_MOESM1_ESM.docx]

**Fig. S1: Electro polymerization of PANI and gold nanoparticle**

A. In-situ voltammograms of a PANI coating measured during its deposition up to 10 cycles with a scan rate of 50mV/s from −0.2 to 0.9 V. B. *In situ*, CV of AuNP scanning from 1.5 V to -0.1 V in 0.5 M H_2_SO_4_ electrolyte containing 0.25 mM HAuCl4 at a scan rate of 50mV/s. inset (single cycle for PANI formation and AuNP deposition)

**Fig. S2: Randle’s circuit used in this study**

**Fig. S3:**

**Validation of the developed sensor in human clinical samples.** y-axis: current density of the sensor after addition of clinical samples, x-axis: study groups. MAT confirmed human sera considered as leptospirosis confirmed sera and Healthy individual sera.

**Table S1: The different elements present in the Nyquist plot at various concentrations of miRNA.**

|  | **R_S_ (Ω cm^2^)** | **CPE (10^-5^)** | **Freq (Hz)** | **R_CT_ (Ω cm^2^)** | **Warburg** | **Ch.Sq (10^-4^)** |
| --- | --- | --- | --- | --- | --- | --- |
| **1 aM** | 88.87 | 7.78 | 0.726 | 451 | 0.00168 | 3.7 |
| **10 aM** | 89.46 | 7.53 | 0.724 | 505 | 0.00163 | 4.49 |
| **100 aM** | 105.2 | 6.48 | 0.725 | 516 | 0.00167 | 2.98 |
| **1 fM** | 98.32 | 5.65 | 0.720 | 579 | 0.00162 | 2.33 |
| **10 fM** | 78.63 | 7.09 | 0.688 | 723 | 0.00163 | 1.38 |
| **100 fM** | 70.73 | 8.86 | 0.664 | 762 | 0.00165 | 1.2 |
| **1 pM** | 73.87 | 8.29 | 0.637 | 908 | 0.00169 | 1.79 |
| **10 pM** | 74.5 | 8.25 | 0.622 | 941 | 0.00166 | 1.98 |
| **100 pM** | 73.01 | 8.28 | 0.656 | 1226 | 0.00163 | 1.39 |
| **1 nM** | 68.83 | 8.68 | 0.655 | 1323 | 0.00168 | 1.95 |
| **10 nM** | 72.07 | 8.05 | 0.660 | 1463 | 0.00170 | 2.14 |
| **50 nM** | 75.79 | 6.88 | 0.672 | 1617 | 0.00165 | 2.04 |
